# Using near-infrared spectroscopy and a random forest regressor to estimate intracranial pressure

**DOI:** 10.1117/1.NPh.9.4.045001

**Published:** 2022-10-11

**Authors:** Filip A. J. Relander, Alexander Ruesch, Jason Yang, Deepshikha Acharya, Bradley Scammon, Samantha Schmitt, Emily C. Crane, Matthew A. Smith, Jana M. Kainerstorfer

**Affiliations:** a Carnegie Mellon University, Department of Biomedical Engineering, Pittsburgh, Pennsylvania, United States; b Carnegie Mellon University, Neuroscience Institute, Pittsburgh, Pennsylvania, United States

**Keywords:** biophotonics, near-infrared spectroscopy, machine learning, intracranial pressure

## Abstract

**Significance:**

Intracranial pressure (ICP) measurements are important for patient treatment but are invasive and prone to complications. Noninvasive ICP monitoring methods exist, but they suffer from poor accuracy, lack of generalizability, or high cost.

**Aim:**

We previously showed that cerebral blood flow (CBF) cardiac waveforms measured with diffuse correlation spectroscopy can be used for noninvasive ICP monitoring. Here we extend the approach to cardiac waveforms measured with near-infrared spectroscopy (NIRS).

**Approach:**

Changes in hemoglobin concentrations were measured in eight nonhuman primates, in addition to invasive ICP, arterial blood pressure, and CBF changes. Features of average cardiac waveforms in hemoglobin and CBF signals were used to train a random forest (RF) regressor.

**Results:**

The RF regressor achieves a cross-validated ICP estimation of $0.937r^{2}$, $2.703\text{-}\mathrm{mm}{\mathrm{Hg}}^{2}$ mean squared error (MSE), and 95% confidence interval (CI) of [−3.064  3.160]  mmHg on oxyhemoglobin concentration changes; $0.946r^{2}$, $2.301\text{-}{\mathrm{mmHg}}^{2}$ MSE, and 95% CI of [−2.841  2.866]  mmHg on total hemoglobin concentration changes; and $0.963r^{2}$, $1.688\;{\mathrm{mmHg}}^{2}$ MSE, and 95% CI of [−2.450  2.397]  mmHg on CBF changes.

**Conclusions:**

This study provides a proof of concept for the use of NIRS in noninvasive ICP estimation.

## 1 Introduction

Intracranial pressure (ICP) is the pressure inside the skull.^1^ Healthy ICP is maintained through a balance of cerebral blood volume, cerebrospinal fluid (CSF), and brain tissue.^2^ Abnormal ICP occurs when an imbalance in one compartment outweighs the compensatory limits of the other two compartments, as explained by the Monro–Kellie doctrine.^2^ An increase in ICP can stem from many issues, including brain bleeds, cerebral edema, a mass lesion such as a brain tumor, and others.^1^

The monitoring of ICP is widely used to guide the treatment of various diseases and illnesses, such as traumatic brain injury (TBI) and hydrocephalus.^3^^,^^4^ TBI, for instance, accounted for over 223,000 hospitalizations in the United States in 2018.^5^ Meanwhile, 1 in every 770 babies born in the United States develop congenital hydrocephalus.^6^ Accurate ICP monitoring is also pertinent to the estimation of cerebral perfusion pressure (CPP), calculated as the difference between mean arterial pressure (MAP) and ICP. CPP, in turn, is used in the gauging of cerebral autoregulation (CA), which defines the brain’s ability to maintain a near constant blood flow when subject to slow changes in blood pressure,^7^^–^^9^ and changes in CPP have been linked to altered neuronal function and neurovascular coupling.^10^^,^^11^ The “gold standard” of ICP measurement is done with an invasive external ventricular drain (EVD). Although the accuracy of an EVD is sensitive to its placement, EVDs have the added benefit of enabling the drainage of excessive CSF.^12^

Although invasive ICP monitoring methods, such as EVDs, microtransducers, and lumbar puncture (LP) manometries, can be precise in their assessment of ICP in patients, they are invasive and not without risks. EVDs, for example, require a coronal burr hole to be drilled and a catheter to be placed near the third ventricle. This can be difficult in some patients, especially in pediatrics for which direct intraparenchymal ICP monitoring has become the norm instead.^13^ EVDs, and microtransducers specifically, have the potential to cause hemorrhage or infection-related complications.^14^ The LP manometries, as a less invasive alternative, measure ICP through CSF pressure in the spinal cord.^15^ LP-based ICP sensing has been shown to agree with EVD-based ICP sensing, but appropriate LP-based sensing depends on 30-min recording periods.^15^^–^^17^ In addition, although LPs are less invasive than EVDs or intraparenchymal probes, LPs not only carry the risk of infection observed with EVDs, but they are also prone to a higher risk of brain herniation and a larger set of contraindications compared with EVDs.^18^^–^^20^

Several noninvasive ICP monitoring methods have been proposed to mitigate the medical risks involved with invasive procedures. Transcranial Doppler (TCD) measures cerebral blood flow (CBF) velocity, from which peak systolic and diastolic flow rates can be extracted. Although some studies have shown that these flow rates correlate with ICP levels, other studies suggest that TCD lacks generalizability.^21^^–^^24^ Likewise, the observation of tympanic membrane displacement coincides with changes in ICP, but the method suffers from poor negative predictive results.^25^^,^^26^ Other noninvasive methods using ultrasound, computed tomography (CT), or magnetic resonance imaging (MRI) tend to either be imprecise, fail to work on some patients, only perform binary ICP-level classification, or not be applicable for continuous ICP monitoring applications. CT-based methods, specifically, suffer from a lack of supporting research that would indicate a predictive use for abnormal CT scans in ICP estimation.^14^^,^^25^ Ultrasound methods, meanwhile, are occasionally afflicted by unwanted artifacts and require a standardized technique across patients for reliable ICP sensing.^14^^,^^25^

Hemodynamic-based methods that use diffuse optical devices, such as near-infrared spectroscopy (NIRS) or diffuse correlation spectroscopy (DCS), have also been used for noninvasive ICP monitoring.^27^^–^^29^ Previously, we have successfully estimated ICP from cardiac waveform features extracted from CBF, measured using DCS.^27^ We have also demonstrated that relative changes in oxyhemoglobin concentrations correlate with relative changes in ICP.^28^ NIRS-based ICP estimation is favorable to other noninvasive ICP monitoring methods, including TCD, CT, MRI, ultrasound, and DCS, due to its potential low cost, user independence, and bedside compatibility for long-term monitoring.

In this work, we explore noninvasive ICP estimation using cardiac waveform features extracted from relative hemoglobin concentration changes, measured using a NIRS device. We compare regression performance on hemoglobin concentration-based signals with regression performance on CBF-based signals taken over approximately the same time periods and across the same set of subjects.

## 2 Materials and Methods

Oxyhemoglobin and deoxyhemoglobin concentration changes along with CBF changes were recorded at various ICP levels with NIRS and DCS across eight nonhuman primates (NHPs). Cardiac pulse waveforms were extracted and processed. Their features were used to train a machine learning algorithm for noninvasive ICP estimation.

### 2.1 Experimental Setup and Signal Extraction

Experimental procedures were approved by the University of Pittsburgh and Carnegie Mellon University’s Institutional Animal Care and Use Committees and complied with the guidelines in the National Institute of Health’s Guide for the Care and Use of Laboratory Animals.

Details of the experimental protocol and data collection have been reported previously.^28^^,^^30^ Briefly, eight anesthetized NHPs (NHPs 1 to 4 *Macaca mulatta* aged 7.9±1.5  years, weighing 9.4±0.7  kg, and NHPs 5 to 8 *Macaca fascicularis* aged 4.2±0.9  years, weighing 5.1±2.1  kg) were used for the experiments. Each NHP was sedated throughout experimentation. Prior to their transport into the experimentation room, the NHPs were sedated with 20-mg/kg ketamine. In some animals, an additional 0.04-mg/kg of atropine and 1-mg/kg diazepam were administered. During the experiment, the monkeys were anesthetized with a combination of 0.6% to 1.5% isoflurane (ISO) and 10- to 25.6-μg/kg/h fentanyl. Additionally, 0.1-mg/kg/h of vecuronium bromide paralytic was administered intravenously. NHPs were ventilated at 0.18 to 0.4 Hz. The ventilation frequency was kept constant for the duration of the experiment for each NHP.

**Fig. 1:**
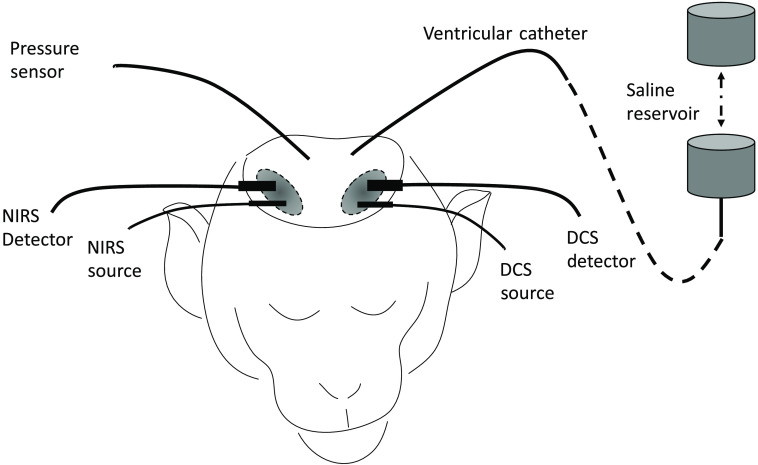
Experimental setup with NIRS and DCS placement on the skull above the right and left prefrontal cortex, respectively. The NIRS setup shows a defined source–detector distance. ICP was measured using a parenchymal pressure sensor. Saline was administered using a ventricular catheter. ICP was regulated by adjusting the height of the saline reservoir.

A frequency-domain NIRS system (OxiplexTS, ISS Inc., United States), as shown in Fig. 1, operating at 690 and 830 nm, was used to measure cerebral hemoglobin concentration changes. The differential pathlength factor (DPF) ratio was held constant between all animals at ${\mathrm{DPF}}_{690}/{\mathrm{DPF}}_{830}=1.1$. Although DPF differences are likely between animals, the cardiac waveform shape is not expected to be influenced significantly. To overcome magnitude differences between animals, signals were normalized in steps described later. Optical probes were placed directly on the skull of the animals, above the right prefrontal cortex. For NHPs 1 to 3, the source detector distance was 2.2 cm, and for NHPs 4 to 8, it was 1.5 cm. The data for this manuscript were based on retrospective analysis. Differences in source detector distances were due to variations in overall experimental designs between animals. Light intensity changes were recorded at a sampling frequency of 50 Hz and converted to changes in oxygenated (ΔHbO), deoxygenated (ΔHb), and total (ΔHbT=ΔHbO+ΔHb) hemoglobin concentrations using the modified Beer–Lambert’s law.^31^ In addition, a DCS probe was placed on the left prefrontal cortex in NHPs 1 to 4 and the right temporal cortex for NHPs 5 to 8, with source detector distances of 2.2 and 1.9 cm, respectively. DCS data from NHP 4 were excluded from further processing due to instrument instabilities. DCS sampling frequency was 100 Hz. Further details of the device are found in Ref. 27 Relative CBF was estimated using a scaled diffusion coefficient measured from the autocorrelation of light intensity changes.^10^ The scaled diffusion coefficient has been shown to correlate with CBF.^32^ CBF was then used for further processing.

Arterial blood pressure (ABP) was recorded with an MPR1 Datalogger (Raumedic Helmbrechts, Germany) using an arterial line placed in the carotid artery. ICP was altered using a catheter (Lumbar catheter, Medtronic, Minneapolis, Minnesota, United States) placed in the lateral ventricle of the brain, with the other end connected to a saline reservoir, as shown in Fig. 1. A change in the height of the reservoir resulted in a pressure change in the head (simulating hydrocephalus). ICP was measured using a parenchymal pressure sensor recorded by the MPR1 Datalogger (Raumedic Helmbrechts, Germany). Both ICP and ABP were recorded at 100 Hz.

Each NHP experiment, lasting ∼22.6  h (std 2.3 h), was split between 7 and 10 separate trials. Each trial was ∼90  min long and corresponded to a particular ICP level, elevated using saline infusion. Induced ICP ranged between 5 and 60 mmHg, with natural ICP fluctuation during experimentation deviating slightly beyond these limits. The distribution of both induced and naturally fluctuating ICP levels was observed to be predominantly between 5 and 30 mmHg. Before ICP elevation, a recording at baseline ICP was also conducted.

### 2.2 Signal Processing

Following the initial data collection, the signal processing procedures closely followed those outlined in Ref. 27 Analog markers in the form of voltage spikes were sent to the auxiliary ports of the DCS, NIRS, and MPR1 Datalogger devices for data alignment. In NHPs 1 to 3, an amplifier circuit was set to register a cardiac pulse when the electrocardiogram (EKG) signal exceeded an empirically determined threshold that defined the R peak of the QRS complex. When an R peak was detected, a synchronization pulse was sent to the MPR1 Datalogger. This signal was sampled at 100 Hz. For NHPs 4 to 8, EKG was measured through a separate device at 1000 Hz. A maximal overlap discrete wavelet transform was run across the EKG signal to enhance the QRS complex features of the signal (the QRS complex represents ventricular polarization). MATLAB’s (MATLAB R2020b, The MathWorks Inc., Natick, Massachusetts, United States) findpeaks function was used to index the time point of the peak of each enhanced QRS complex. The respective indices represent the R peak of the QRS complex. During data collection, occasional laser instabilities were observed. Time points in the NIRS- and DCS-based signals, for which instabilities were visually observed, were removed. For NHP 4, the DCS-based CBF signal showed consistent instability across all trials. As a result, NHP 4 was not included in the ICP estimation analysis of CBF. All signals not originally sampled at 50 Hz (ABP, ICP, CBF, and QRS complex peak indices) were downsampled to 50 Hz to match the sampling frequency of the NIRS signal.

To reduce noise in the NIRS and DCS signals, 120 consecutive cardiac pulses were averaged, and an average cardiac waveform (ACPW) was extracted. Because the heart rate of the animals varied, 120 pulses corresponded to between 39 and 78 s across all animals. The ICP values during the 120 pulses were also averaged. The 120-pulse averaging window was moved 20 pulses at a time (resulting in an 83.3% window-to-window overlap) along the entire signal of each trial. All ACPWs were normalized in time and amplitude. With the help of spline interpolation, the length of each ACPW was normalized to 66 data points, corresponding to 1.32 s. ACPW length was measured between two consecutive diastoles. The height of each ACPW (representing the amplitude of ΔHbO, ΔHbT, and CBF) was normalized to between 0 and 1. Normalization of the x- and y-axis removed ACPW length as a feature and thus removed heart rate as a feature of the waveform. We removed heart rate as a feature because heart rate changes can be independent of ICP.^33^^–^^35^

To remove outliers, a z-score rejection method was applied to all ACPWs. For each trial, a z-score was calculated across all averaged pulses in the trial. This averaged z-score was then compared against each ACPW. Any ΔHbO, ΔHbT, or CBF ACPW with a z-score greater than 3 was rejected.

**Fig. 2:**
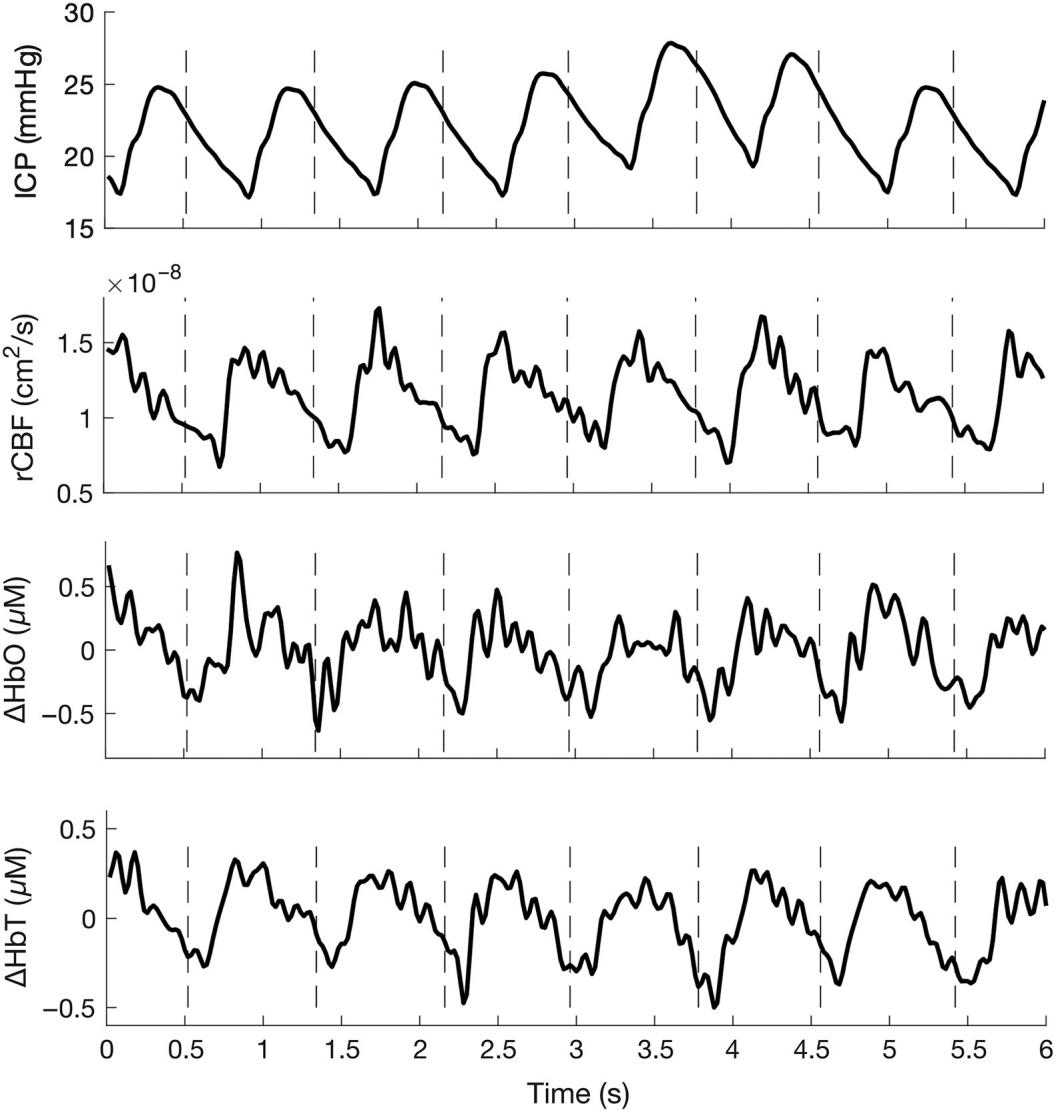
Filtered time trace examples of ICP, CBF, ΔHbO, and ΔHbT signals. Dashed lines indicate QRS complex peaks.

A Kalman filter was then used to further improve signal quality. The adaptive filter was applied to the ACPWs of each signal for each trial. Each ACPW was compared with the ideal trial pulse produced by the Kalman filter. ACPWs were corrected based on their error from the ideal pulse. In the correction method, the Kalman filter’s parameters defined the weight given to the Kalman-produced ideal pulse and the weight given to the calculated ACPW-to-ideal-pulse error. These parameters were set empirically. The output of the Kalman filter procedure was a set of high signal-to-noise ratio (SNR) ACPWs with feature morphology reflecting changes in ICP. ACPWs reflecting ICP values above 30 mmHg were removed due to their scarcity. Figure 2 shows examples of 50 Hz ΔHbO, ΔHbT, ICP, and CBF signals. A total of 19,000 ΔHbO and 19,258 ΔHbT ACPWs sampled from 8 NHPs, along with 17,322 CBF ACPWs sampled from 7 of the same 8 NHPs were used for feature extraction. All ACPWs represented an ICP range of 0 to 30 mmHg.

### 2.3 Feature Extraction

**Fig. 3:**
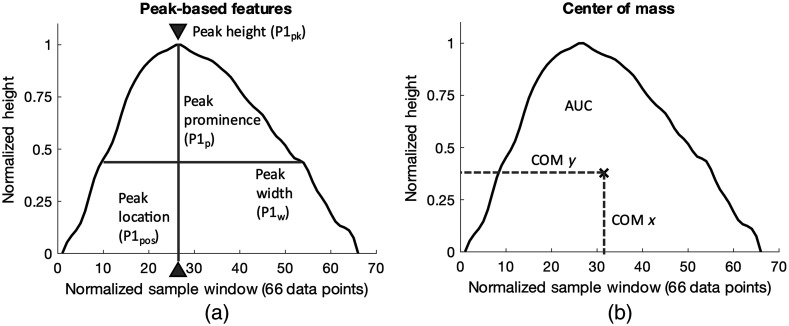
(a) Peak-based features, including peak height (${P1}_{\mathrm{pk}}$) (normalized to 1), peak width (${P1}_{w}$), peak prominence (${P1}_{p}$), and peak location (${P1}_{\mathrm{pos}}$), for a typical ΔHbO ACPW. (b) x- and y-coordinates of the center of mass of the same ACPW. MAP was used as an additional feature, not shown here.

Defining and extracting physiologically relevant features from the processed ACPWs provides the basis for estimating ICP. In our previous work, features of the CBF waveform included the percussion, tidal, and dicrotic peaks of the ACPWs.^27^ These features were not generally discernable from the NIRS-based hemoglobin concentration signals. As such, the feature set and their extraction method were modified, as shown in Fig. 3. Figure 3 also shows an example of a ΔHbO ACPW.

MATLAB’s findpeaks function was used to obtain the peak height (${P1}_{\mathrm{pk}}$: normalized to 1 and used as a measure for model noise), position (${P1}_{\mathrm{pos}}$), prominence (${P1}_{p}$), and width (${P1}_{w}$) of the ACPWs.

The x- and y-coordinates of the center of mass (centroid) of the waveform, as shown in Fig. 3(b), defined as COMx and COMy, were also extracted from individual ACPWs and used as features. This was done by turning each ACPW into a polygon and calculating its centroid using the polyshape and centroid functions in MATLAB. We hypothesized that COMx, which describes waveform skewness, is related to blood pressure and ICP. A similar reasoning motivated COMy. The area under the curve (AUC) of the waveform was also incorporated as a feature, as was MAP. Similar to ICP, MAP was calculated over each 120-pulse window. The feature engineering resulted in eight human-interpretable and observable features for each processed ΔHbO, ΔHbT, and CBF ACPW. If a feature was undetected, its value was set to 0, but it was still used.

### 2.4 Machine Learning

Each of the three ACPW datasets was randomly sampled into five cross validation (CV) sets of 80% training and 20% testing. For each CV set, all animals were included. Random sampling ensured that learning became NHP and trial independent, while CV allowed us to alleviate overfitting. Python’s scikit-learn toolbox’s random forest (RF) regression algorithm was used as the ICP estimator.^36^ The RF algorithm, explained in detail by Ref. 37, learns a set number of decision trees on a randomly sampled subset of the features using a randomly sampled subset of the training data with replacement (bootstrapping). A total of 100 trees, or estimators, were learned using this bootstrapping method. Every tree received 50% of the features and 80% of the dataset for training. All other hyperparameters were kept as default, defined in the scikit-learn documentation.^36^ These hyperparameters were chosen empirically to maximize performance while mitigating overfitting. The hyperparameter decision-making process used a random search to gauge approximate hyperparameter ranges, after which a per-parameter and joint-parameter optimization procedure followed. Random search hyperparameter tuning is a common method used for optimizing machine learning models.^38^ One important parameter was the number of estimators, or trees. Each of the 100 trees splits its subset of data until all leaves are pure—to max depth. Each tree receives four randomly sampled features for learning. Gini impurity was used as the measure of node split quality.^39^ For each CV split (fold), the RF algorithm was trained on the training split and tested on the testing split. During testing, estimated ICP values (${\mathrm{ICP}}_{\mathrm{est}}$) were compared with invasively measured ICP values (${\mathrm{ICP}}_{\mathrm{inv}}$). ${\mathrm{ICP}}_{\mathrm{inv}}$ values were used as ground truth labels. The performance of the model was quantified using the coefficient of determination ($r^{2}$), mean squared error (MSE), and 95% confidence interval (CI).

## 3 Results

### 3.1 Waveform Features and RF Regressors

We have previously found that the CBF ACPW broadens and skews to the right when ICP increases.^27^ We qualitatively observed similar trends in the ΔHbO and ΔHbT ACPWs.

**Fig. 4:**
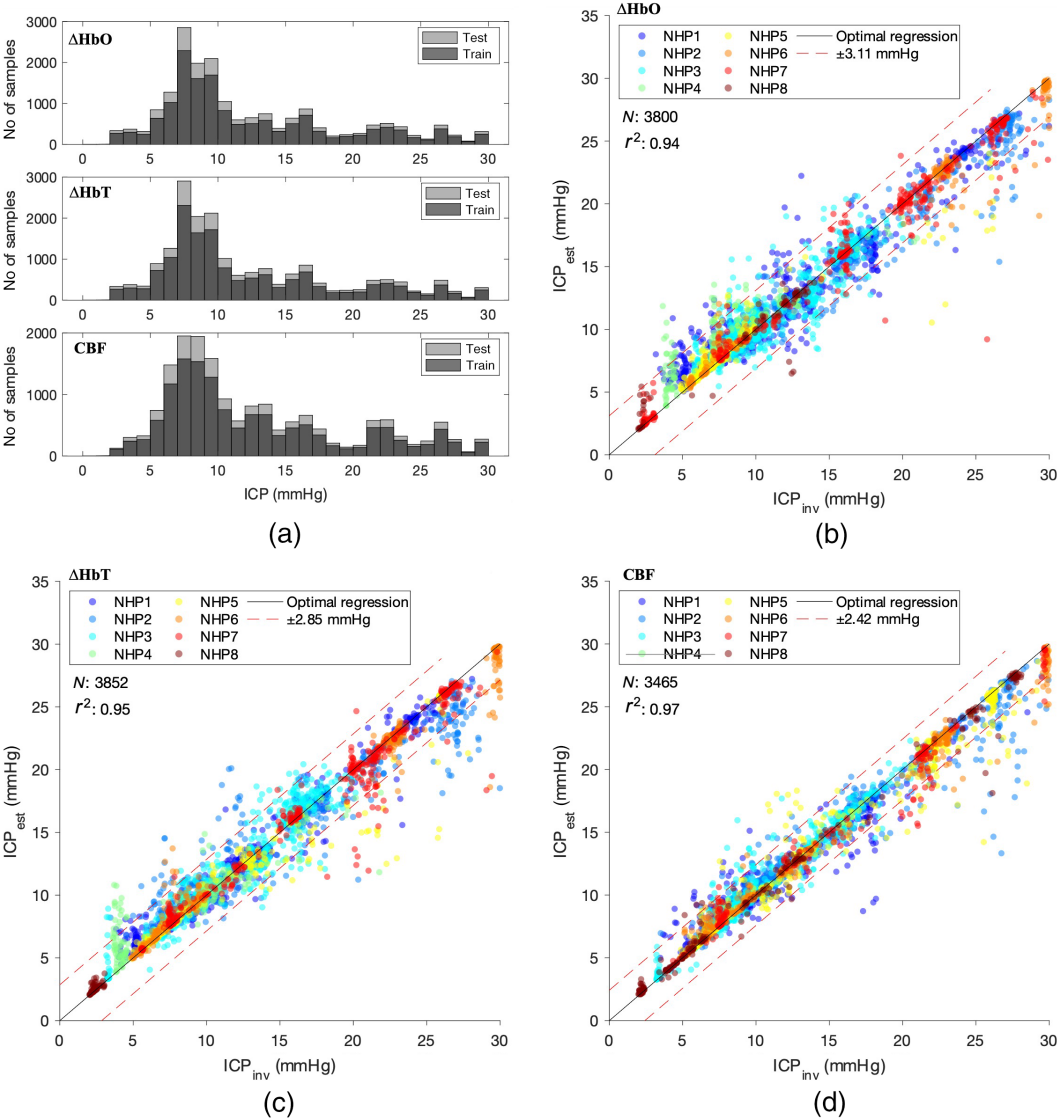
(a) Histogram of ICP distribution for both train and test sets across all three modalities. More data were available at lower ICP values, especially between 5 and 10 mmHg. (b)–(d) Correlation plots for ΔHbO, ΔHbT, and CBF. Strong $r^{2}$ for all methods suggests that the model performs well on ICP estimation. Estimation performance drops for higher ICP values across all three modalities due to a lower availability of high ICP data for training.

Using an RF regressor with modified hyperparameters, we evaluated the ICP estimator’s performance on NIRS-derived ACPWs (ΔHbO and ΔHbT) against its performance on DCS-based ACPWs (CBF). Although the CBF-based waveforms showed more morphological features compared with the ΔHbO- and ΔHbT-based waveforms, the features chosen were the same between all three modalities used. Within the 0 to 30 mmHg range of ICP values, available training and testing data were skewed toward lower ICP values, as shown in Fig. 4(a).

To compare performance differences between the three different types of signals, the coefficient of determination ($r^{2}$) and MSE were used as evaluation metrics, along with a 95% CI. A fivefold CV was performed individually on the ΔHbO, ΔHbT, and CBF datasets. We found a mean fold $r^{2}=0.937$ (averaged over five folds), $r^{2}=0.946$, and $r^{2}=0.963$ for the ACPW types ΔHbO, ΔHbT, and CBF, respectively, with a fivefold $r^{2}$ standard deviation of $r_{\mathrm{std}}^{2}=0.003$ (ΔHbO), $r_{\mathrm{std}}^{2}=0.004$ (ΔHbT), and $r_{\mathrm{std}}^{2}=0.002$ (CBF). We further found a mean fold MSE=2.703, 2.301, and $1.688\;{\mathrm{mmHg}}^{2}$ with a fivefold standard deviation of ${\mathrm{MSE}}_{\text{std}}=0.133$, 0.163, and $0.131\;{\mathrm{mmHg}}^{2}$, respectively. Our results indicate that CBF waveforms perform better than NIRS-derived parameters in terms of ICP extraction, but ICP can still be quantified within an MSE of $<3\;{\mathrm{mmHg}}^{2}$ when using hemoglobin-based waveforms.

The correlation plots between ${\mathrm{ICP}}_{\mathrm{est}}$ and ${\mathrm{ICP}}_{\mathrm{inv}}$ for all three signals are shown in Figs. 4(b)–4(d). All three ICP extractions show a good correlation between estimated and invasively measured ICP. Outliers were more common at higher ICP values for which less data were available for training, as shown in the histograms of Fig. 4(a).

**Fig. 5:**
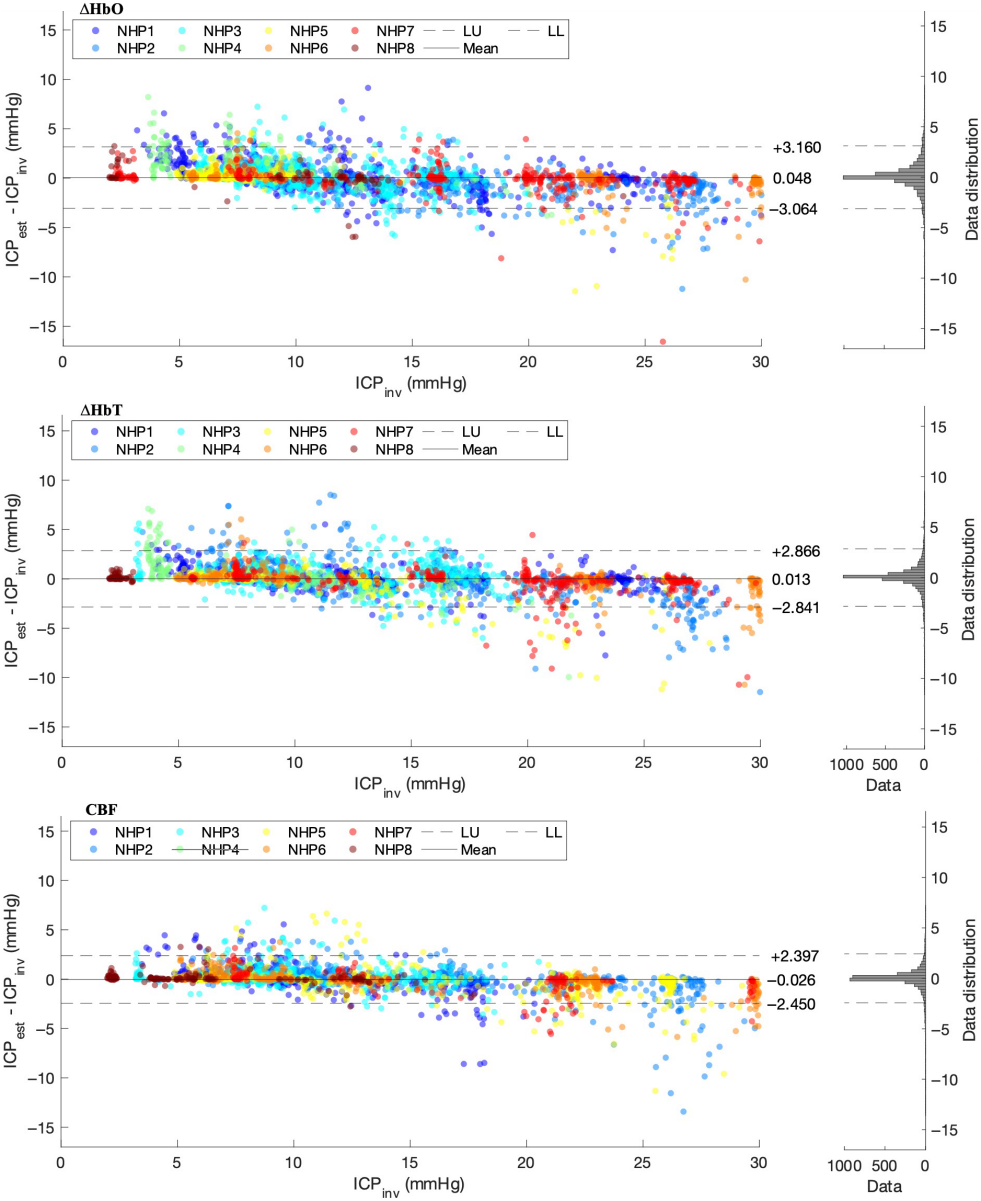
Bland–Altman plots for ΔHbO, ΔHbT, and CBF. Histograms show the distribution of datapoints across the Bland–Altman plot.

A set of three Bland–Altman plots, indicating the level of agreement between ${\mathrm{ICP}}_{\mathrm{est}}$ and ${\mathrm{ICP}}_{\mathrm{inv}}$, are shown in Fig. 5 and further confirm the clear fit of our regression model to the invasively measured ICP. The plots show a 95% CI of agreement between ${\mathrm{ICP}}_{\mathrm{est}}$ and ${\mathrm{ICP}}_{\mathrm{inv}}$ of [−3.064  3.160]  mmHg with a mean of 0.048 mmHg for ΔHbO, a 95% CI of [−2.841  2.866]  mmHg with a mean of 0.013 mmHg for ΔHbT, and a 95% CI of [−2.450  2.397]  mmHg with a mean of −0.026  mmHg for CBF.

**Fig. 6:**
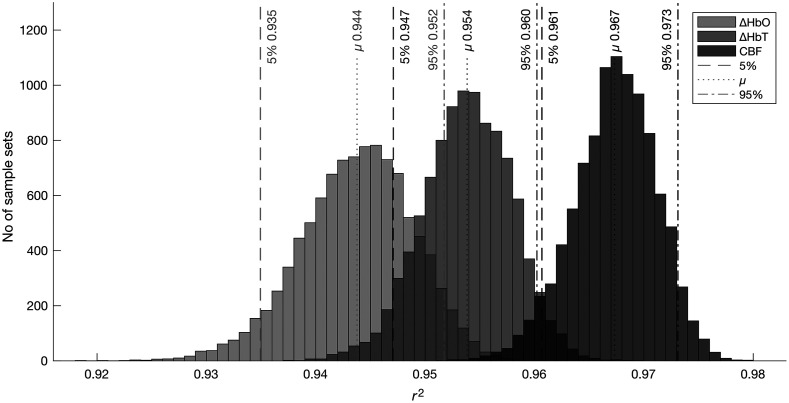
Histograms of bootstrapped $r^{2}$ scores for all three modalities. Bootstrapping used 50% of test samples drawn 10,000 times with replacement. The test data from the best performing CV split, per modality, are used. This results in sample mean $r^{2}$ slightly above the mean CV $r^{2}$ reported. $r^{2}$ is calculated for each set of samples across all three modalities (ΔHbO, ΔHbT, and CBF). A significance level ≤0.05 is used. CBF statistically outperforms both NIRS methods. NIRS methods are still comparable to CBF in overall performance.

Table 1 summarizes the results across all three modalities. When considering the performance of ΔHbO against ΔHbT and CBF, we find that ΔHbT performs slightly better than ΔHbO. ΔHbO and ΔHbT have a 0.009 difference in fivefold mean $r^{2}$ and a $0.402\;{\mathrm{mmHg}}^{2}$ difference in fivefold mean MSE. CBF performs better than the two NIRS-based methods with an increase of 0.025 fivefold mean $r^{2}$ and a $0.402\;{\mathrm{mmHg}}^{2}$ decrease in fivefold mean MSE against ΔHbO. The 95% CI metric across the modalities echoes this difference in model fit performance. Using bootstrap analysis in which 50% of test samples were randomly drawn 10,000 times with replacement and a respective $r^{2}$ was calculated for each set of samples, we found that CBF performs statistically better than either of the NIRS methods (significance level (alpha)≤0.05). Despite this, NIRS performs comparatively well against DCS, along with being cheaper and easier to use. Distributions of $r^{2}$ scores with 5%, mean (μ), and 95% thresholds are shown in Fig. 6.

**Table 1:** Error metrics across the three modalities.

Modality	$r^{2}$	MSE	$r_{\mathrm{std}}^{2}$	${\mathrm{MSE}}_{\mathrm{std}}$	95% CI	Mean
CBF	0.963	1.688	0.002	0.131	[−2.450 2.397]	−0.026
ΔHbT	0.946	2.301	0.004	0.163	[−2.841 2.866]	0.013
ΔHbO	0.937	2.703	0.003	0.133	[−3.064 3.160]	0.048

**Fig. 7:**
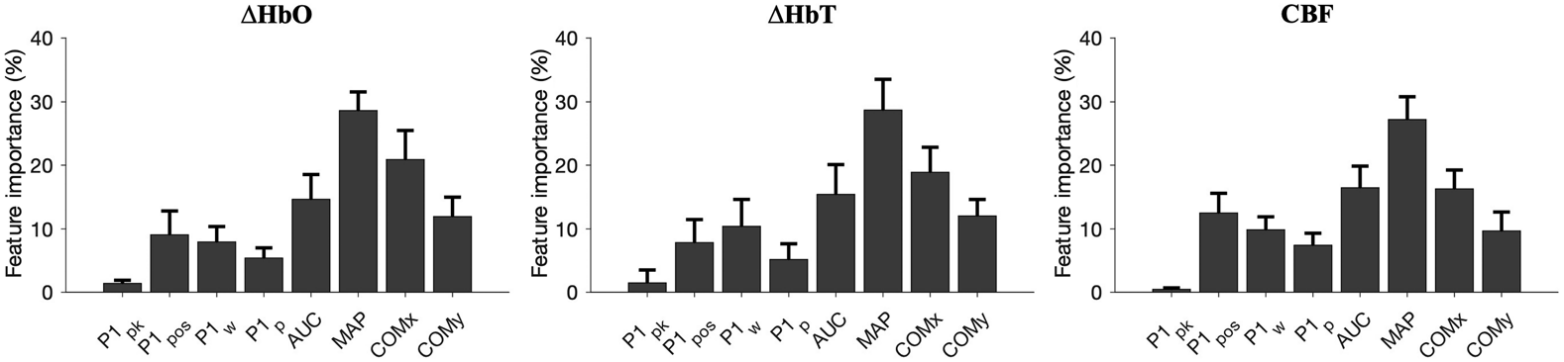
Comparison of feature importance across ΔHbO, ΔHbT, and CBF. MAP is strongest across all three modalities. AUC and COMx are relevant for estimation, and peak-based features (${P1}_{\mathrm{pos}}$, ${P1}_{w}$, and ${P1}_{p}$) are more important in CBF-based ICP estimation compared with the hemoglobin-based methods. It is also important to note that ${P1}_{\mathrm{pk}}$ is low for all methods, especially for CBF. This suggests that the model does not learn irrelevant features.

For all three modalities, the feature relevance contributing to ICP extraction was evaluated. The relative magnitude of feature importance is shown in Fig. 7. We found that MAP and COMx were the most relevant features used in the training of each estimator tree. AUC, COMx, and MAP accounted for ∼15%, ∼20%, and ∼28% of node splits per tree, respectively. Meanwhile, COMy represented ∼11% of node splits, whereas waveform peak position represented ∼9%. The remaining three features represented <7% of node splits per tree. The standard deviation of features used between trees is shown by the error bars of each feature bar. AUC importance for ΔHbO, ΔHbT, and CBF was approximately the same at around 15% to 18%, whereas peak-related features (peak location, width, and prominence) were more important for CBF compared with the NIRS methods. COMx and COMy were less important for ΔHbT and CBF compared with ΔHbO. MAP was the most important feature across all modalities, with the level of importance being approximately the same. For ΔHbT, MAP represented ∼29% of splits per tree compared with ∼28% for ΔHbO and ∼27% for CBF. The importance of the height of the peak, which was normalized, is used as a proxy to measure the importance of an unrelated peak. The apparent relevance of this unrelated peak is highest for ΔHbO and lowest for CBF, suggesting that the model learns slightly more uncorrelated patterns on the ΔHbO dataset compared with the datasets of the other modalities.

A more evenly balanced dataset was also tested for ΔHbO. Balancing was performed by randomly removing half of all ICP values between 5 and 10 mmHg. This balanced dataset had ∼23% less data than the skewed dataset. The balanced dataset led to a marginally worse performance compared with the skewed dataset and was not studied further for either ΔHbT or CBF. For ΔHbO, $r^{2}$ dropped by 0.6% from 0.937 to 0.932, MSE increased by 22.1% from 2.703 to $3.299\;{\mathrm{mmHg}}^{2}$, and mean 95% CI increased by [12.3% 11.5%] from [−3.064  3.160] to [−3.441  3.523]  mmHg with a 14.6% change in mean from 0.048 to 0.041 mmHg. Because MAP is a dominant feature for all three signals used, we evaluated whether MAP can be removed to make DCS and NIRS standalone methods for ICP extraction. When MAP was removed as a training feature, the importance of the four peak features was increased, and the importance of AUC and COMx was maintained. With MAP removed, performance fell to $r^{2}=0.838$, $\mathrm{MSE}=6.971\;{\mathrm{mmHg}}^{2}$, and 95% CI of [−4.960  4.984]  mmHg with a mean of 0.012 mmHg for ΔHbO; $r^{2}=0.865$, $\mathrm{MSE}=5.791\;{\mathrm{mmHg}}^{2}$, and 95% CI of [−4.556  4.700]  mmHg with a mean of 0.072 mmHg for ΔHbT; and $r^{2}=0.916$, $\mathrm{MSE}=3.778\;{\mathrm{mmHg}}^{2}$, and 95% CI of [−3.785  3.756]  mmHg with a mean of −0.014  mmHg for CBF. These results indicate that the performance is improved with MAP as an added feature, but ICP can still be quantified to <∼5  mmHg for hemoglobin-based estimators and <∼4  mmHg for CBF-based estimators.

## 4 Discussion

The goal of this work was to demonstrate that noninvasive ICP measurements are possible when using hemoglobin concentration changes as measured with NIRS. Here we showed that, although the NIRS-based estimate is worse than one obtained using DCS, ICP could still be estimated within ∼3  mmHg in eight NHPs for an ICP range of between 0 and 30 mmHg. The performance of the estimator used in this manuscript improves on that in our previous work with DCS devices^27^ due to the larger dataset and the refined regression model. The results suggest that NIRS could be an alternative to DCS in terms of noninvasive ICP.

### 4.1 Dataset

In building an NHP-based dataset of ICP-dependent ACPW features, it was observed that ΔHbO and ΔHbT cardiac pulse waveforms followed a canonical cardiac pulse arch shape. Using 120-pulse averaging and Kalman filtering improved SNR to the extent that meaningful feature extraction could be performed. The number of pulses averaged over was set to 120 to include enough pulses for appropriate waveform and ICP averaging. Averaging too many pulses would likely lead to lower ICP-to-waveform-feature correlation, whereas not averaging enough pulses would not remove enough noise in the signal waveforms.

Despite selecting only ICP values up to 30 mmHg, the distribution of data was not equal for all ICP values, as shown in the histogram in Fig. 4(a). We found that balancing the dataset did not significantly improve ICP estimation. There appeared to be a more even distribution of outliers in ICP estimation between low and high ICP (0 to 30 mmHg) in the balanced dataset, compared with the skewed dataset (data not shown). Meanwhile, feature importance was not significantly different between the skewed and balanced data. It can be argued that the unbalanced dataset was more likely to cause overfitting to lower ICP values and to artificially inflate performance. Although possible, we do not have enough data points at higher (>20  mmHg) ICP values to quantify this.

### 4.2 Preprocessing and Filtering

To appropriately leverage the raw signals extracted from each NHP, signal preprocessing and filtering were required. The goal was to provide the feature extraction method with low-noise signals while not overfiltering or losing valuable information. Clean signals lead to relevant feature-value-to-ICP mappings for the machine learning model to learn.

Tuning of the signal preprocessing and filtering parameters was done empirically across the cardiac waveform averaging, waveform normalization, z-score rejection, and Kalman filtering methods. 120 consecutive pulses were found to be a suitable averaging amount to reduce signal to noise, whereas a 20-pulse averaging window shift struck a balance between obtaining ample amounts of data for training and testing while reducing data replication. Waveform normalization, z-score rejection, and Kalman filtering all worked to remove data outliers while maintaining a feature distribution that supported model generalization.

### 4.3 Feature Engineering

An investigation into feature importance and performance, as shown in Fig. 7, suggests that MAP on its own, though relevant, is not the only feature driving decision tree learning. This gives weight to our other chosen features, such as AUC and COMx. We also found poor correlation between the raw values of MAP, AUC, and COMx with ICP. This suggests that a feature on its own is not a good indicator of ICP, giving strength to our approach of combining several features in a machine learning method to accurately estimate ICP. Interpretable feature engineering is an important part of clinically relevant and understandable machine learning tools. Explainable models ensure that conclusions made by the model are based on appropriate decisions.^40^

Extracting specific features from data may improve model generalizability for small sample sizes. The FDA’s *Good Machine Learning Practice for Medical Device Development: Guiding Principles*^41^ highlights the importance of human interpretability of models and their outputs in clinical settings. As such, the features that we engineered are more interpretable when compared with machine-engineered features sometimes used in complex machine learning and neural network-based methods.^29^ We also found that our engineered features expressed themselves relatively predictably across modalities.

### 4.4 RF Regression

In our previous work on DCS-based ICP extraction, we showed a cross-validated similarity between estimated ICP and invasively measured ICP of $r^{2}=0.91$, an MSE of 3.3 mmHg, and a 95% CI of [−3.7  3.7]  mmHg. Here we compared NIRS-derived waveforms to DCS waveforms for ICP extraction and achieved a cross-validated similarity between DCS-based ICP estimation and invasively measured ICP of $r^{2}=0.96$, MSE of 1.7 mmHg, and 95% CI of [−2.450  2.397]  mmHg. This is an improvement to our previous work. The performance increase was likely due to using more data and a revised machine learning method. Compared with Ruesch et al.’s 14,121 ACPWs across 5 NHPs,^27^ our CBF dataset contained 17,322 ACPWs sampled from 7 NHPs. The ICP distributions were similar. Regarding the ICP estimators, Ruesch et al.^27^ used a bagged ensemble of trees with subsampling of data. Unlike our RF model, which was chosen for its versatility, robustness, resistance to noise, and ability to manage unbalanced datasets,^42^^–^^45^ the bagged tree regressor of Ruesch et al.^27^ did not randomly subsample features for each tree in the ensemble. In an RF regression, the random sampling of features ensures that no one tree receives the full set of features for learning. This combats overfitting and improves performance.

RF also have a relatively low risk of overfitting with an increase in estimators due to their use of multiple weak and unpruned learners.^46^ Each tree in the RF ensemble does overfit to the data and the features that it receives, but the averaged result of the ensembled trees produces a regression prediction that is low in variance and bias. Ultimately, this means that the more trees that are used in the ensemble, the less likely the model is to overfit. More trees, however, increase computational cost. Compared with Ruesch et al.’s 1000 trees,^27^ we achieved comparable results with only 100 trees. This means that our approach is more computationally efficient with comparable results.

It is important to note that the signal processing, feature extraction, and training methods used on the three signal types explored in this paper (ΔHbO, ΔHbT, and CBF) were the same and that the features were specifically engineered with NIRS-based signals in mind. DCS-based (CBF) ICP estimation with these specific features was relatively more dependent on peak-based features than the ΔHbO and ΔHbT ICP estimation, but not to the same extent as observed in Ruesch et al.^27^ Meanwhile, features such as AUC, COMx, and COMy were important across the modalities. MAP was the defining feature for all signal types, with varying degrees of relative importance compared with other features. This suggests that the chosen features are potentially useful across signal and sensor modalities. CBF waveforms, however, do carry more waveform-related information compared with waveforms extracted using blood volume changes. Thus, waveform-based features are more relevant for CBF compared with ΔHbO and ΔHbT. The features chosen for this work may therefore not be optimized for CBF, and further improvement in ICP extraction may be possible.

### 4.5 Feature Differences Across NHPs

Occasionally observed differences in the morphology of the same waveform feature across subjects are likely due in part to influences on the NHPs’ natural autoregulation and their abilities to manage varying ICPs. Because ISO gas, commonly used during anesthesia, is known to suppress CA,^47^^,^^48^ we used <1% to avoid anesthesia induced autoregulation impairment.^30^ However, changes in ICP led to CPP levels outside the autoregulatory range.^8^^,^^9^^,^^30^ The Windkessel effect—the elasticity of arteries that moderates blood pressure changes during a cardiac cycle—may also be a factor in observed morphological differences. Studies show that conduit arteries harden with age.^49^ The stiffening of cerebral arteries results in an increased pulse pressure per stroke volume and a higher systolic pressure. This effect has primarily been studied in humans, but the effect may be similar in the NHPs used and could therefore contribute to inconsistent morphology of volume-dependent cardiac pulse waveforms between NHPs of different ages. Our youngest NHP was 3 years old, and our oldest was 10 years old. Macaques reach sexual maturity at 3 to 5 years (REFS) and can live to 25 years or more in captivity.^50^^,^^51^

Small differences in probe and catheter placement between NHPs were also likely contributors to observed feature dissimilarity and variance between ${\mathrm{ICP}}_{\mathrm{inv}}$ and ${\mathrm{ICP}}_{\mathrm{est}}$. Although care was taken in the placement of the NIRS device on the skull of the NHPs, small variations did exist between subjects. In addition, ICP sensor and ventricular catheter placement varied slightly due in part to distinctive subject physiologies.

### 4.6 Future Work

One avenue of future study along model selection could investigate probabilistic RF (PRF). Reis et al.^52^ showed that, compared with normal RF, PRFs improved classification by 10% on noisy features and 30% on noisy labels. This model may also be useful for regression. Natural continuations of this study would include large-scale human validation. Due to the relatively small sample size, we did not attempt a leave-one-out analysis. All animals were represented in both the training and the testing sets. A meaningful assessment of the generalizability of our ICP estimator to unseen data would be facilitated by a larger dataset.

An additional future consideration could evaluate the importance of the location of MAP estimation. In this manuscript, we measured MAP from the carotid artery. An alternative approach may the use of a blood pressure cuff, which hypothetically provides a sufficient MAP reading to be used with our approach. This, however, has not yet been evaluated. If successful, an MAP reading using a blood pressure cuff would ease clinical translation of our noninvasive ICP estimation method.

## 5 Conclusions

This work demonstrates that ICP can be accurately predicted using an RF regressor trained on cardiac pulse waveform features extracted from hemoglobin-based signals using NIRS or DCS. It also shows that ICP-dependent cardiac pulse waveform morphology is discernible in hemoglobin-based signals sampled at 50 Hz. We compared the performance of our algorithm on ΔHbO-, ΔHbT-, and CBF-based data and found comparable accuracy when using features optimized for hemoglobin concentration changes. Our approach is EKG and MAP dependent, both of which are regularly used and measured in clinical settings. An increased NHP sample size, human trials and a balanced representation of ICP values are important to validating our findings for the clinical setting.

## Code, Data, and Materials Availability

Code and data are available upon request.
